# Exploring the Association of Systolic Blood Pressure and Intracranial Pressure Variability and Subarachnoid Hemorrhage Patient Outcomes

**DOI:** 10.3390/jcm15103748

**Published:** 2026-05-13

**Authors:** Stephanie Cardona, Saad Pirzada, Jane Quackenbush, Joshua Olexa, Abbey Kim, Yiting Lin, Quincy K. Tran

**Affiliations:** 1 Department of Critical Care Medicine, Baptist Health System, Miami, FL 33144, USA; 2 Research Associate Program in Emergency Medicine and Critical Care, Department of Emergency Medicine, University of Maryland School of Medicine, Baltimore, MD 21201, USA; 3 University of Maryland School of Medicine, Baltimore, MD 21201, USAqtran@som.umaryland.edu (Q.K.T.); 4 Department of Neurosurgery, University of Maryland School of Medicine, Baltimore, MD 21201, USA; 5 Department of Emergency Medicine, University of Maryland School of Medicine, Baltimore, MD 21201, USA; 6 Program in Trauma, University of Maryland School of Medicine, Baltimore, MD 21201, USA

**Keywords:** blood pressure variability, discharge disposition, intracranial pressure variability, mortality, neurocritical care, subarachnoid hemorrhage

## Abstract

**Background**: Subarachnoid hemorrhage (SAH) results from extravasation of blood into the subarachnoid space and is associated with high morbidity and mortality. This study aimed to compare systolic blood pressure variability (SBPV) and intracranial pressure variability (ICPV) in three 8 h intervals during the first 24 h after hospital admission and investigate their associations with discharge disposition and in-hospital mortality. **Methods**: We retrospectively reviewed charts of adult patients with spontaneous, non-traumatic SAH admitted for at least 24 h from 2016 to 2020. Hourly measurements were recorded for both systolic blood pressure (SBP) and intracranial pressure (ICP), and SBPV and ICPV were measured using successive variation (SV) and standard deviation (SD). **Results**: A total of 240 patients were included (mean age 57 ± 14.2 years, 64.6% female); 40 (16.7%) died. Univariate analyses showed higher SBP-SV (22.7 ± 13.8) in the first 8 h to be significantly associated with mortality (*p* = 0.028) and not being discharged home (*p* = 0.022), compared to those who survived (17.6 ± 7.5) or were discharged home (16.7 ± 5.5). Multivariate logistic regression did not show an association between SBPV and outcomes of interest. **Conclusions**: Greater early SBPV was associated with mortality in univariate analysis but was not independently predictive after adjustment for clinical severity, suggesting it reflects underlying physiologic instability rather than an independent prognostic factor in SAH.

## 1. Introduction

Subarachnoid hemorrhage (SAH) results from the extravasation of blood into the subarachnoid space. It is the third most common cerebrovascular disorder after acute ischemic stroke and intracerebral hemorrhage, and is associated with high morbidity and mortality [1]. Most cases of SAH are traumatic; about 80% of spontaneous SAH results from aneurysmal rupture, which is associated with high rates of mortality [1,2,3]. Recent research and clinical interest has been focused on the association between fluctuations of specific neurophysiologic measures such as blood pressure variability (BPV) and intracranial pressure variability (ICPV) and clinical outcomes in patients with SAH [4,5].

For decades, aggressive management of high blood pressure (BP) has been foundational in the initial treatment of patients with SAH; current guidelines recommend lowering systolic blood pressure (SBP) to equal or less than 140–160 mmHg (prior to securing the aneurysm) [1,6,7,8]. Accumulating evidence over more recent years suggests that BPV is also critical and adversely related to outcomes for this group of patients [5,6,8,9]. Although the exact mechanism for why increased BPV worsens SAH patient outcomes is unknown, it is hypothesized that BPV may be a reflection of poor cerebral autoregulation [4,9,10].

On the other hand, the scant literature on ICPV suggests that greater variation in ICPV predicts better outcomes for patients with aneurysmal subarachnoid hemorrhage (aSAH) [4]. Although the available literature evaluating ICPV in SAH is limited, it suggests a consistent association with clinical outcomes. In a cohort study by Kirkness et al., greater early variability in intracranial pressure was associated with 6-month functional outcomes, establishing ICPV as a potential prognostic marker [11]. More recent investigations by Wettervik et al. have found that lower ICPV was consistently associated with unfavorable outcomes, including increased risk of delayed cerebral ischemia and worse functional recovery, suggesting that reduced variability may reflect impaired cerebrovascular reactivity [4]. On the other hand, higher ICPV correlated with lower cerebrovascular resistance and more preserved cerebral hemodynamics [10,12]. Collectively, these data support ICPV as a potential prognostic marker in SAH, where diminished variability appears to be linked to cerebrovascular dysfunction and poorer clinical outcomes.

Given the limited literature on systolic blood pressure variation (SBPV) and ICPV and its association with outcomes in patients with SAH, especially in time intervals that include the acute and hyperacute periods of SAH treatment, our study sought to compare SBP and ICPV in three separate 8 h intervals during the first 24 h of admission (the first, second, and third 8 h intervals). We hypothesized that SBPV and ICPV would be greatest in the first 8 h. Additionally, we explored both SBPV and ICPV and their association with outcomes in patients with SAH and compared the predictive power of these neurophysiologic measures. We hypothesized that greater SBPV is associated with worse patient outcomes and ICPV, given our variable timeframe of 8 h and that our outcome measures only encapsulate the first 24 h, would not be associated with patient outcomes.

## 2. Materials and Methods

This retrospective study included all adult patients (age ≥ 18) who experienced a non-traumatic, spontaneous SAH, and were admitted to the University of Maryland Medical Center for a minimum of 24 h and monitored via an external ventricular drain (EVD). The study period was from 2016 to 2020.

### 2.1. Outcome Measures

The primary outcome of interest was in-hospital mortality. A secondary outcome of interest was discharge home, which was used as a surrogate for neurologic functional outcome. Prior studies have shown interrater discrepancies > 23% when Cerebral Performance Category (CPC) scale or modified Rankin Scale (mRS) was used in a retrospective manner [13,14,15]. Therefore, discharge location was used instead to assess for neurological functional outcome.

### 2.2. Independent Variables

All patient data was obtained through the Electronic Medical Record (EMR). Some variables of interest included patients’ SBP, ICP, Glasgow Coma Scale (GCS), and Hunt and Hess scale grading (classification tool which helps assess severity and prognosis of patients with aSAH) (Table 1 and Table 2). At our institution, patients with SAH undergo BP and ICP measurements at least hourly for the first 24 h. We sought to explore SBPV and ICPV through these hourly measurements, in three 8 h intervals: the first 8 h, the second 8 h, and the third 8 h. These variations can be calculated in many ways; for this study, we measured both SBPV and ICPV through successive variation (SV) and standard deviation (SD). The successive variation in systolic blood pressure (SBP-SV) is measured by taking the absolute difference between consecutive systolic blood pressure measurements and averaging these values. The standard deviation of systolic blood pressure (SBP-SD) is measured by taking the mean of the absolute difference in each systolic blood pressure measurement. The same applies for ICPV but using ICP instead of SBP measurements.

### 2.3. Data Collection and Management

All team members involved in data collection were trained by the principal investigator (PI). In the training process, all team members who collected data were first given a sample data collection of 10 patient charts and were required to achieve a minimum of 90% agreement with PI data before proceeding. All data was compiled into Microsoft Excel spreadsheet (Microsoft Corp, Seattle, WA, USA). After data collection, a team member randomly checked 20% of the collected data to ensure an interrater agreement of at least 90% was maintained.

### 2.4. Data Analysis

We employed descriptive analyses such as sample size (N) and percentage (%) for categorical variables and interquartile ranges (IQR), means, and SD for continuous variables. We employed Mann–Whitney U test and independent sample T-test to compare continuous data between groups, including mortality (alive vs. dead) and discharge status (discharged home vs. not discharged home); Chi-Square test was used for categorical variables.

Given that SBP-SV was statistically significant in the deceased group, we sought to further examine the relation between SBP-SV in the first 8 h interval and mortality by performing a univariate probit logit analysis. To further probe the discriminatory abilities of SBPV and ICPV in the varying 8 h intervals for mortality and discharge home, an area under the receiver operating curve (AUROC) analysis was done. The AUROC analysis was parameterized for mortality such that 1 = dead and 0 = alive. For discharge home, it was such that 1 = not discharged home and 0 = discharged home. An AUROC < 0.5 indicated no discriminatory ability, 0.5–0.6 was failed, 0.6–0.7 was poor, 0.7–0.8 was fair, 0.8–0.9 was good, and 0.9–1.0 was excellent.

Data analysis was conducted using both Minitab version 18.1 (Minitab Corp, State College, PA) and R version 4.4.2 (R Core Team, 2022).

## 3. Results

**Figure 1:**
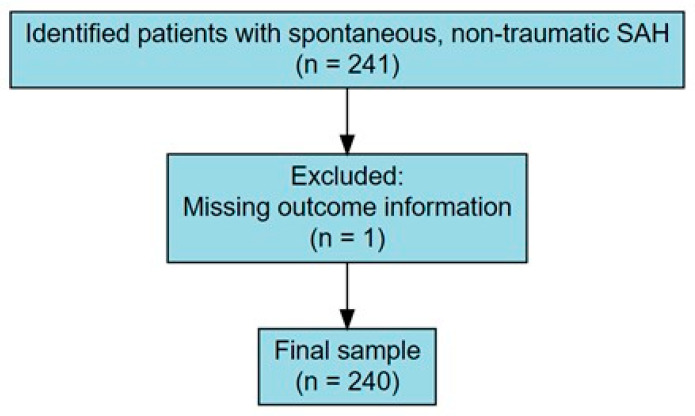
Flow diagram of patient inclusion and exclusion. Abbreviations: SAH, subarachnoid hemorrhage.

Our study identified 241 patients with spontaneous, non-traumatic SAH admitted to our institution (Figure 1); one was excluded due to missing outcome information. Out of the 240 included, 200 were aneurysmal and 40 were non-aneurysmal SAH. Of the 240, 155 were female (64.6%), and the mean age was 57 (±14.2). The average age of patients discharged home was 49 (±12.2), which was 10.5 years younger than those not discharged home (59 ± 13.8; *p* < 0.001). Overall, 40 patients died (16.7%) (Table 1). Upon admission, the median GCS score was 9 [IQR 6–14]. The admission GCS for patients who survived (9, [IQR 6.3–14]) was on average 4 points greater than the admission GCS of patients who did not survive (5, [IQR 3–7], *p* < 0.001). A total of 101 (42.1%) of the patients underwent a craniectomy; of these, 91 (46%) survived and 10 (25%) died (*p* = 0.01). The average Hunt and Hess score among all patients was 3.25 (±1.16). Patients who survived had a score of 3.07 (±1.1) while those who died had a significantly higher score of 4.24 (±0.94; *p* < 0.001).

Increased SBP-SV among SAH patients within the first eight hours of admission (first interval) was significantly greater in both patients who died and were not discharged home. Specifically, SBP-SV for patients who died was 22.7 (±13.8), which was 5.1 points greater than those who survived (17.6 ± 7.5, *p* = 0.028). Among those not discharged home, the mean SBP-SV of 19.0 (±9.8) was 2.4 points greater than those discharged home (16.7 ± 5.5, *p* = 0.022). No intervals for ICPV were significantly different regarding mortality or disposition. However, ICP-SV in the first eight hours approached a significant difference in patients who were not discharged home (4.4 ± 3.9) compared to those discharged home (3.5 ± 2.0; *p* = 0.054).

### 3.1. Probit Analysis: Mortality

**Figure 2:**
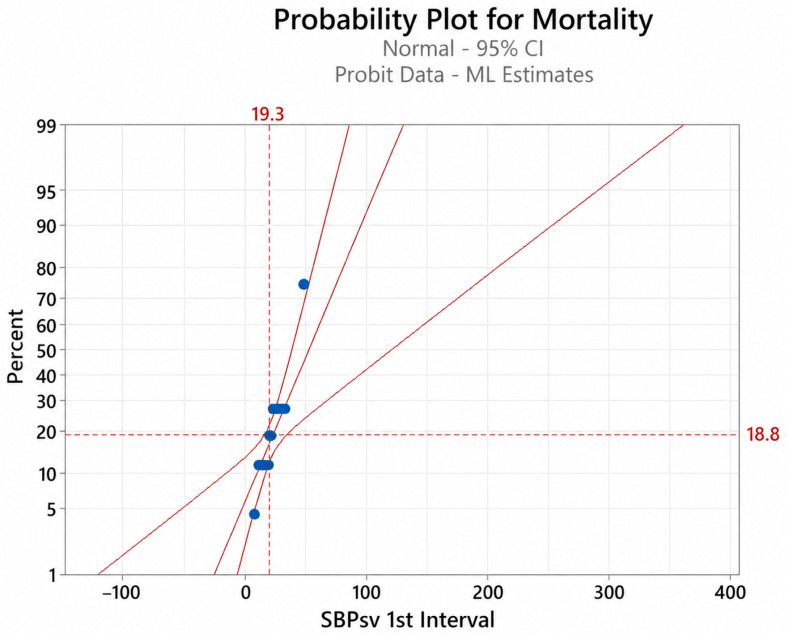
Probit analysis—1st 8 h interval. Abbreviations: CI = confidence interval; ML = maximum likelihood; SBPsv = successive variation in systolic blood pressure.

Probit analysis was used to investigate the probability of mortality through a clinical predictor, specifically the continuous variable of SBP-SV. The univariate probit analysis found that SBP-SV, in the first eight hours, significantly predicted the outcome of mortality (*p* < 0.01). Furthermore, the results of our model suggest an SBP-SV of approximately 19 was associated with mortality in around 19% of our patient population (Figure 2).

### 3.2. Receiving Operating Curve (ROC) Analysis: Mortality and Discharge Home

**Figure 3:**
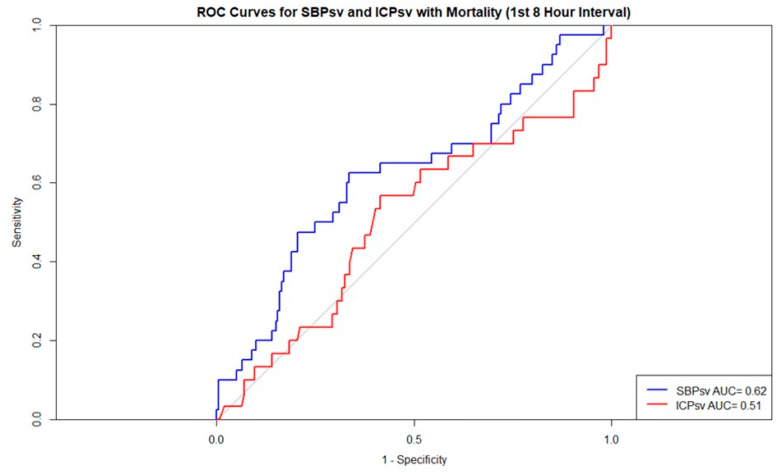
ROCs for mortality—1st 8 h interval. Abbreviations: AUC = area under the curve; ICPsv = successive variation in intracranial pressure; ROC = receiver operating characteristic; SBPsv = successive variation in systolic blood pressure.

**Figure 4:**
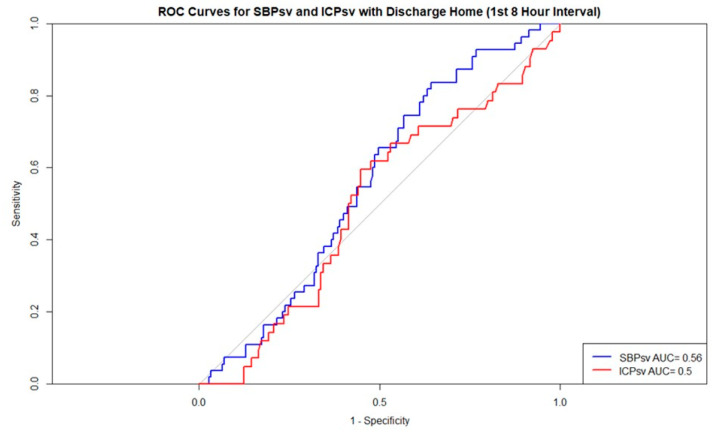
ROCs for discharge home—1st 8 h interval. Abbreviations: AUC = area under the curve; ICPsv = successive variation in intracranial pressure; ROC = receiver operating characteristic; SBPsv = successive variation in systolic blood pressure.

In the ROC used to predict mortality in the first interval, according to the standard interpretations of ROCs, ICP-SV, with an area under the curve (AUC) of 0.51, failed to predict mortality whereas SBP-SV, with an AUC of 0.62, demonstrated poor discrimination (Figure 3) [16]. All other time intervals in regard to mortality and discharge home for both SBP-SV and ICP-SV failed at predicting outcomes (Figure 4; Supplemental Figures S1 and S2).

### 3.3. Multivariable Logistic Regression

A multivariable logistic regression model was performed adjusting for age, admission GCS, and Hunt & Hess score, due to large differences in these variables accross outcome groups.

In the core model adjusted for age, admission GCS, and Hunt & Hess score, first-interval SBP-SV was not independently associated with in-hospital mortality (adjusted OR per 5-unit increase 1.08, 95% CI 0.86–1.36; *p* = 0.519; N = 214) or not being discharged home (adjusted OR per 5-unit increase 1.08, 95% CI 0.79–1.49; *p* = 0.613; N = 214). In full continuous-covariate models including age, admission GCS, sodium, platelet count, glucose, lactate, INR, heart rate, 24 h fluid balance, and Hunt & Hess score, first-interval SBP-SV remained non-significant for mortality (adjusted OR per 5-unit increase 1.24, 95% CI 0.87–1.74; *p* = 0.230; N = 159) and not being discharged home (adjusted OR per 5-unit increase 1.21, 95% CI 0.78–1.87; *p* = 0.389; N = 159).

## 4. Discussion

Previous research suggests greater ICPV in short-term variations, specifically variations between 55 and 15 s, is associated with decreased risk of cerebral delayed ischemia in aSAH patients in the early post-ictal phase and early vasospasm phase [4]. However, these associations in the early phases did not emerge when longer ICP variations were utilized, such as 30 min and 4 h. Instead, associations between ICPV in 30 min and 4 h parameters emerged in the late vasospasm phase (days 6.5–10). The short-term fluctuation in ICPV and its association with better outcomes is hypothesized to arise from the possible indication of healthier cerebral vessels with less atherosclerosis and cerebral vasospasm [17]. Although the literature suggests higher short-term ICPV is associated with better clinical outcomes in aSAH patients in the early post-ictal and early vasospasm phase, our results showed no significant difference in ICPV in any of the time intervals for either mortality or discharge home. One possibility for the difference in results is that our ICPV was parameterized in 8 h intervals, longer than any of the time intervals in the aforementioned study, where the longest parameter was 4 h. Moreover, our outcomes of interest were mortality and discharge home and were not divided into different phases, such as in the study conducted by Wettervik et al., with phases of 1–3 days, 3–6.5 days, and 6.5–10 days [4,10]. Differences in ICPV parameters and differences in outcome times do not allow for direct comparison to the current literature. Although ICP-SV in the first interval approached statistical significance (*p* = 0.054), this did not meet conventional thresholds, and therefore should be interpreted cautiously.

In univariate analyses, greater SBPV, measured as SBP-SV in the first 8 h interval, was associated with mortality and discharge disposition. However, these associations did not persist after adjustment for age and baseline neurologic severity, suggesting that the observed relationships were likely confounded by underlying disease severity rather than reflecting an independent effect of SBPV. This finding is consistent with the concept that hemodynamic variability may serve as a marker of physiologic instability in patients with more severe hemorrhage, rather than a direct mediator of outcome.

Prior studies have reported associations between increased SBPV and adverse outcomes in aSAH. For example, Cai et al. demonstrated that higher SBPV within the first 24 h following endovascular treatment was associated with mortality and unfavorable neurologic outcome at 6 months [17]. Similarly, Kirkness et al. found that variability in blood pressure over longer time intervals was associated with worse clinical outcomes, whereas shorter-term variability was associated with improved survival, suggesting differing physiologic implications across time scales [11]. While our unadjusted findings are directionally consistent with these reports, the lack of independent association after adjustment in our cohort suggests that these relationships may be mediated by differences in baseline clinical severity and physiologic reserve. One potential explanation for the observed univariate associations is that early BPV reflects a critical period of physiologic instability, during which complications such as rebleeding and secondary brain injury are more likely to occur [9,18,19]. However, given that these associations were not independent after adjustment, SBPV may be better understood as a surrogate marker of early disease severity and treatment intensity rather than a direct contributor to these complications.

Importantly, SBP-SV demonstrated poor discriminatory performance for mortality, and ICP-SV showed no meaningful discriminatory ability, further limiting their utility as standalone prognostic markers. These findings underscore the complexity of physiologic variability in SAH and highlight the importance of accounting for confounding variables when evaluating potential prognostic indicators.

Our study has several limitations. This was a single-center retrospective analysis, which may limit generalizability. However, our cohort reflects a high-acuity population managed with standardized, contemporary neurocritical care protocols, supporting the broader relevance of the observed physiologic relationships. Additionally, SBP and ICP data were collected hourly and aggregated into 8 h intervals, which may obscure shorter-term fluctuations that have been shown to carry physiologic significance. Differences in ICPV parameterization compared to prior studies further limit direct comparisons. We did not assess long-term functional outcomes, such as 6-month Glasgow Outcome Scale or modified Rankin Scale scores, and instead used discharge disposition as a surrogate, which may be influenced by non-clinical factors including socioeconomic status, insurance coverage, and the availability of social support. Finally, the substantial overlap in SBP-SV values between outcome groups likely contributed to the observed poor discriminative performance [20,21].

## 5. Conclusions

In this cohort of patients with SAH, greater SBPV in the early phase was associated with mortality in univariate analyses; however, this relationship was not independently significant after adjustment for age and baseline neurologic severity. These findings suggest that early SBPV should be interpreted as a marker of underlying physiologic instability rather than an independent prognostic factor. Further multicenter studies are needed to clarify the role of BPV in SAH and to determine whether it represents a modifiable target.

## Figures and Tables

**Table 1:** Demographics and clinical characteristics of patients with SAH and between-group differences according to clinical outcomes.

	All Patients (N = 240)	Alive	Dead/Hospice	Diff.	*p*	Not D/C Home	D/C Home	Diff.	*p*
Demographics									
Age, years, mean (SD)	57 (14.2)	57 (14)	60 (14.9)	−3.4	0.19	59 (13.8)	49 (12.2)	10.47	<0.001
Gender, N (%)									
Male	85 (35.4)	71 (35.5)	14 (35)	0.05	0.81	69 (37.3)	16 (29)	0.09	0.21
Female	155 (64.6)	129 (64.5)	26 (65)	−0.05	1	116 (62.7)	39 (71)	−0.08	0.34
Past medical history									
Hypertension, N (%)	155 (64.6)	125 (63)	30 (75)	−0.13	0.10	124 (67)	31 (56)	0.11	0.16
Diabetes, N (%)	42 (17.5)	35 (18)	7 (18)	0	1	33 (18)	9 (16)	0.01	0.80
Home medications									
Any anticoagulation, N (%)	15 (6.25)	12 (6)	3 (7)	−0.02	0.74	13 (7)	2 (4)	0.04	0.28
Any anti-platelet, N (%)	60 (24.5)	47 (24)	13 (33)	−0.09	0.26	51 (28)	9 (16)	0.11	0.06
Glasgow Coma Scale									
At admission, median [IQR]	9 [6–14]	9 [6.25–14]	5 [3–7]	4	<0.001	7 [5–13]	13 [7–14]	−3	<0.001
At 24 h, median [IQR]	9 [7–13]	10 [8–14]	5 [3.25–7]	4	<0.001	8 [6–10]	14 [10–15]	−4	<0.001
Admission laboratory values									
Sodium, mean (SD)	139.5 (4.3)	139.3 (4.0)	140.6 (5.4)	−1.21	0.18	139.8 (4.4)	138.8 (3.9)	0.94	0.13
Platelet count, mean (SD)	234.9 (78.1)	236 (66.8)	229 (120)	6.9	0.73	232 (81.7)	245 (63.3)	−13	0.22
Glucose, mean (SD)	168.5 (61.8)	167 (62.3)	176 (59.1)	−9.1	0.38	169.4 (58.1)	165.5 (73.3)	3.9	0.72
Lactate, mean (SD)	2.9 (6)	2.8 (6.5)	3.2 (1.8)	−0.43	0.49	3 (6.6)	2.2 (1)	0.79	0.16
INR, mean (SD)	1.1 (0.13)	1.1 (0.12)	1.1 (0.14)	−0.05	0.04	1.1 (0.1)	1 (0.1)	0.06	<0.001
Other clinical variables									
Mechanical ventilation, N (%)	217 (90.4)	177 (89)	40 (100)	−0.11	0.03	180 (98)	37 (67)	0.31	<0.001
Any seizure in hospital, N (%)	44 (18.3)	34 (17)	10 (25)	−0.08	0.28	37 (20)	7 (13)	0.07	0.18
Craniectomy, N (%)	101 (42.1)	91 (46)	10 (25)	0.21	0.01	79 (43)	22 (40)	0.03	0.72
Hunt & Hess score, mean (SD)	3.3 (1.2)	3.1 (1.1)	4.2 (0.94)	−1.17	<0.001	3.5 (1.2)	2.6 (0.9)	0.86	<0.001
Vasospasm, N (%)	137 (57.1)	125 (62.5)	12 (30)	0.34	<0.001	107 (60)	30 (55)	0.06	0.44

Abbreviations: Diff. = difference; D/C = discharged; INR = international normalized ratio; IQR = interquartile range; SD = standard deviation.

**Table 2:** Fluid balance, in-hospital treatments, and neurophysiologic measures in patients with SAH and in-group differences according to clinical outcomes.

	All Patients (N, %)	Alive	Dead/Hospice	*p*	D/C Home	Not D/C Home	*p*
In-hospital clinical variables							
Balance of fluid in/out (SD)	543 (1976)	451 (1859)	891 (2355)	0.23	263 (1272)	620 (2127)	0.13
Any anti-seizure medication, N (%)	44 (18.3)	34 (17)	10 (25)	0.33	7 (12.8)	37 (20)	0.31
Any hyperosmolar therapy, N (%)	73 (30.4)	49 (24.5)	24 (60)	<0.001	9 (16.4)	64 (34.6)	0.02
Blood products administration							
None, N (%)	178 (0.7)	147 (0.7)	31 (0.8)	0.74	41 (0.7)	137 (0.7)	1
Any, N (%)	61 (25.4)	52 (26)	9 (22.5)	0.79	14 (25.5)	47 (25.4)	1
Platelets, N (%)	56 (0.2)	48 (0.2)	8 (0.2)	0.73	13 (0.2)	43 (0.2)	1
pRBCs, N (%)	2 (0.01)	2 (0.01)	0 (0)	1	0 (0)	2 (0.01)	1
FFP, N (%)	3 (0.01)	2 (0.01)	1 (0.02)	1	1 (0.02)	2 (0.01)	1
Blood pressure variability							
SBPmax, mean (SD)	181.96 (27)	180.54 (23.2)	188.95 (40.4)	0.21	180.76 (20.8)	182.31 (28.5)	0.66
SBPmin, mean (SD)	96.78 (20.7)	96.99 (20.6)	95.75 (21.3)	0.73	97.04 (21.1)	96.71 (20.6)	0.92
SBP-SD (first 8 h), mean (SD)	18.03 (7.2)	17.49 (6.3)	20.71 (10.6)	0.07	17.16 (5.8)	18.28 (7.6)	0.25
SBP-SD (second 8 h), mean (SD)	14.72 (6.7)	14.37 (5.6)	16.49 (10.4)	0.22	14.23 (5.8)	14.87 (6.9)	0.50
SBP-SD (third 8 h), mean (SD)	13.79 (7.4)	13.6 (6.3)	14.7 (11.7)	0.58	12.68 (5.9)	14.11 (7.8)	0.15
SBP-SV (first 8 h)	18.49 (9.0)	17.64 (7.5)	22.74 (13.8)	0.03	16.65 (5.5)	19.04 (9.8)	0.02
SBP-SV (second 8 h)	15.80 (9.1)	15.57 (8.4)	16.95 (11.8)	0.48	15.05 (7.4)	16.02 (9.5)	0.43
SBP-SV (third 8 h)	15.25 (7.6)	15.26 (7.8)	15.20 (7.1)	0.96	14.66 (7.1)	15.42 (7.8)	0.51
Intracranial pressure variability							
ICP-SD (first 8 h), mean (SD)	3.68 (3.3)	3.42 (2.3)	5.00 (6.3)	0.18	3.13 (1.9)	3.84 (3.6)	0.09
ICP-SD (second 8 h), mean (SD)	3.06 (2.0)	3.03 (1.8)	3.20 (2.8)	0.70	2.94 (1.9)	3.09 (2.0)	0.65
ICP-SD (third 8 h), mean (SD)	3.05 (1.9)	2.94 (1.8)	3.64 (2.7)	0.14	2.94 (1.9)	3.08 (2)	0.65
ICP-SV (first 8 h), mean (SD)	4.19 (3.6)	4.02 (3.1)	5.04 (5.5)	0.33	3.52 (2)	4.39 (3.9)	0.05
ICP-SV (second 8 h), mean (SD)	3.64 (2.4)	3.60 (2.2)	3.82 (3.2)	0.71	3.68 (2.4)	3.63 (2.4)	0.89
ICP-SV (third 8 h), mean (SD)	3.67 (2.4)	3.60 (2.3)	4.05 (2.9)	0.40	3.54 (2.2)	3.71 (2.5)	0.66

Abbreviations: D/C = discharged; FFP = fresh frozen plasma; ICP = intracranial pressure; ICP-SD = standard deviation of intracranial pressure; ICP-SV = successive variation in intracranial pressure; pRBCs = packed red blood cells; SBP = systolic blood pressure; SBP-SD = standard deviation of systolic blood pressure; SBP-SV = successive variation in systolic blood pressure; SD = standard deviation.

## Data Availability

The raw data supporting the conclusions of this article will be made available by the authors on request.

## Supplementary Materials

The following supporting information can be downloaded at: https://www.mdpi.com/article/10.3390/jcm15103748/s1, Figure S1: ROCs for Mortality—2nd and 3rd 8 h interval; Table S1: Regression Table and Goodness-of-Fit Tests; Figure S2: ROCs for Discharge Home—2nd and 3rd 8 h interval. Table S2: Multivariable Logistic Regression Models.
