# Supplementary material for: Exploring the Association of Systolic Blood Pressure and Intracranial Pressure Variability and Subarachnoid Hemorrhage Patient Outcomes
Source: J Clin Med. 2026 May 13;15(10):3748. doi: 10.3390/jcm15103748 (PMC13207548; doi:10.3390/jcm15103748)
Supplement: Supplementary file 1 [file jcm-15-03748-s001.zip › jcm-4250575-supplementary.pdf]

Supplementary Table S1. Regression Table and Goodness-of-Fit Tests

Regression Table

Variable	Coefficient	Standard Error	Z	*p*
Constant	-1.547	0.225	-6.87	0.000
SBP-SV (First 8 hour interval)	0.0298	0.0103	2.90	0.004

p = p-value; SBP-SV = successive variation of systolic blood pressure; SE = standard error; Z = Z-score.

Goodness-of-Fit Tests

Method	Chi-Square	DF	*p*
Pearson	240.77	237	0.420
Deviance	207.35	237	0.918

DF = degrees of freedom; p = p-value

Supplementary Table S2. Multivariable Logistic Regression Models

Model	N	Events	Variable	Adjusted OR	95% CI	p-value	Model AUC
Core adjusted model: mortality	214	33	SBP-SV (first 8 hours), per 5-unit increase	1.08	0.86–1.36	0.519	0.865
Core adjusted model: mortality	214	33	Age, per 10-year increase	1.32	0.93–1.86	0.121	0.865
Core adjusted model: mortality	214	33	Admission GCS	0.69	0.58–0.83	<0.001	0.865
Core adjusted model: mortality	214	33	Hunt & Hess score	1.78	1.10–2.86	0.018	0.865

Full continuous model: mortality	159	28	SBP-SV (first 8 hours), per 5-unit increase	1.24	0.87–1.74	0.230	0.889
Full continuous model: mortality	159	28	Age, per 10-year increase	1.48	0.97–2.25	0.069	0.889
Full continuous model: mortality	159	28	Admission GCS	0.65	0.51–0.84	<0.001	0.889
Full continuous model: mortality	159	28	Admission sodium	1.04	0.93–1.16	0.489	0.889
Full continuous model: mortality	159	28	Platelet count, per 50-unit increase	1.02	0.78–1.34	0.873	0.889
Full continuous model: mortality	159	28	Admission glucose, per 50-unit increase	0.95	0.59–1.52	0.822	0.889
Full continuous model: mortality	159	28	Admission lactate	1.06	0.98–1.14	0.177	0.889
Full continuous model: mortality	159	28	Admission INR	16.11	0.52–501.10	0.113	0.889
Full continuous model: mortality	159	28	Admission heart rate, per 10-unit increase	1.07	0.79–1.45	0.642	0.889
Full continuous model: mortality	159	28	24-hour fluid balance, per 1 L increase	1.43	1.06–1.93	0.020	0.889
Full continuous model: mortality	159	28	Hunt & Hess score	1.4	0.75–2.61	0.288	0.889
Core adjusted + ICP-SV model: mortality	188	30	SBP-SV (first 8 hours), per 5-unit increase	1.02	0.80–1.30	0.883	0.904
Core adjusted + ICP-SV model: mortality	188	30	ICP-SV (first 8 hours), per 1-unit increase	0.93	0.83–1.04	0.182	0.904
Core adjusted + ICP-SV model: mortality	188	30	Age, per 10-year increase	1.42	0.96–2.09	0.078	0.904
Core adjusted + ICP-SV model: mortality	188	30	Admission GCS	0.63	0.50–0.78	<0.001	0.904
Core adjusted + ICP-SV model: mortality	188	30	Hunt & Hess score	2.43	1.34–4.41	0.003	0.904

Core adjusted model: not discharged home	214	161	SBP-SV (first 8 hours), per 5-unit increase	1.08	0.79–1.49	0.613	0.889
Core adjusted model: not discharged home	214	161	Age, per 10-year increase	2.54	1.78–3.62	<0.001	0.889
Core adjusted model: not discharged home	214	161	Admission GCS	0.68	0.58–0.80	<0.001	0.889
Core adjusted model: not discharged home	214	161	Hunt & Hess score	1.54	1.01–2.36	0.045	0.889
Full continuous model: not discharged home	159	125	SBP-SV (first 8 hours), per 5-unit increase	1.21	0.78–1.87	0.389	0.908
Full continuous model: not discharged home	159	125	Age, per 10-year increase	2.89	1.75–4.77	<0.001	0.908
Full continuous model: not discharged home	159	125	Admission GCS	0.66	0.53–0.82	<0.001	0.908
Full continuous model: not discharged home	159	125	Admission sodium	1.05	0.91–1.21	0.531	0.908
Full continuous model: not discharged home	159	125	Platelet count, per 50-unit increase	0.67	0.46–0.95	0.027	0.908
Full continuous model: not discharged home	159	125	Admission glucose, per 50-unit increase	0.87	0.56–1.34	0.516	0.908
Full continuous model: not discharged home	159	125	Admission lactate	1.04	0.94–1.15	0.442	0.908
Full continuous model: not discharged home	159	125	Admission INR	1549.62	2.72–882852.16	0.023	0.908
Full continuous model: not discharged home	159	125	Admission heart rate, per 10-unit increase	0.85	0.62–1.15	0.285	0.908

Full continuous model: not discharged home	159	125	24-hour fluid balance, per 1 L increase	1.16	0.88–1.54	0.294	0.908
Full continuous model: not discharged home	159	125	Hunt & Hess score	1.89	1.04–3.43	0.038	0.908
Core adjusted + ICP-SV model: not discharged home	188	141	SBP-SV (first 8 hours), per 5-unit increase	1.03	0.73–1.43	0.884	0.892
Core adjusted + ICP-SV model: not discharged home	188	141	ICP-SV (first 8 hours), per 1-unit increase	1.12	0.92–1.36	0.272	0.892
Core adjusted + ICP-SV model: not discharged home	188	141	Age, per 10-year increase	2.43	1.68–3.51	<0.001	0.892
Core adjusted + ICP-SV model: not discharged home	188	141	Admission GCS	0.69	0.59–0.82	<0.001	0.892
Core adjusted + ICP-SV model: not discharged home	188	141	Hunt & Hess score	1.65	1.04–2.61	0.032	0.892

Supplementary Figure S1. ROC Curves for Mortality 2nd and 3rd 8-hour interval

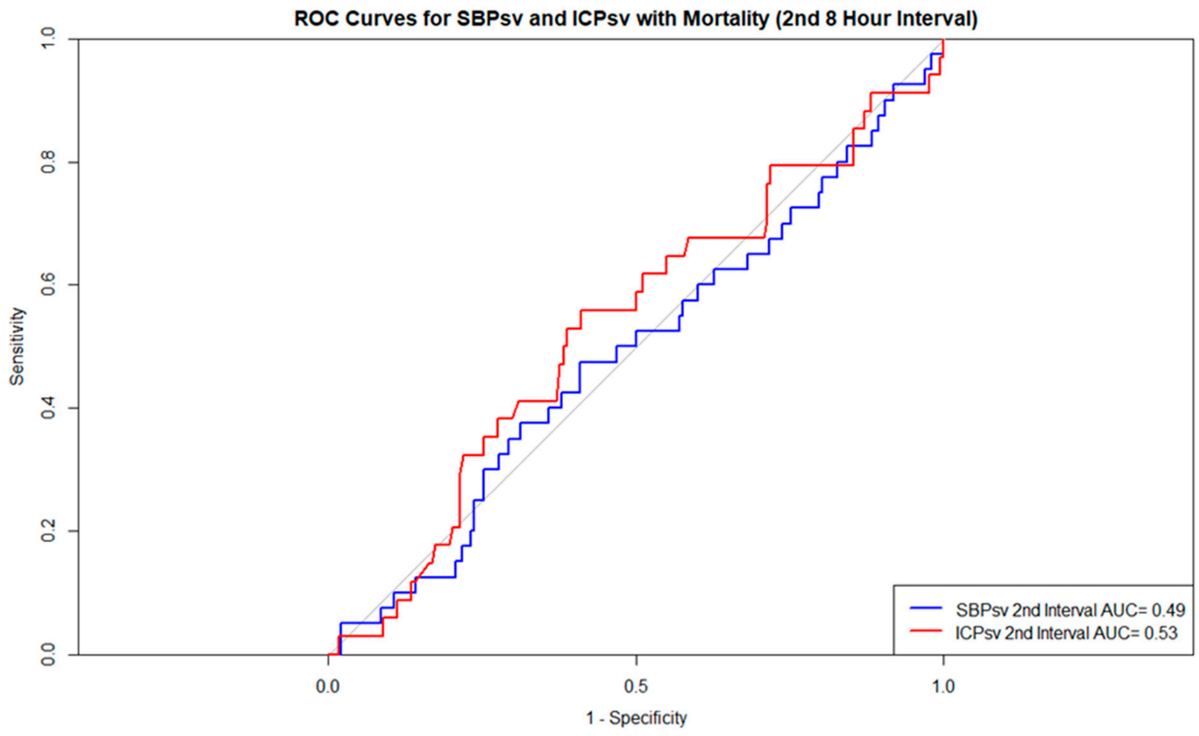

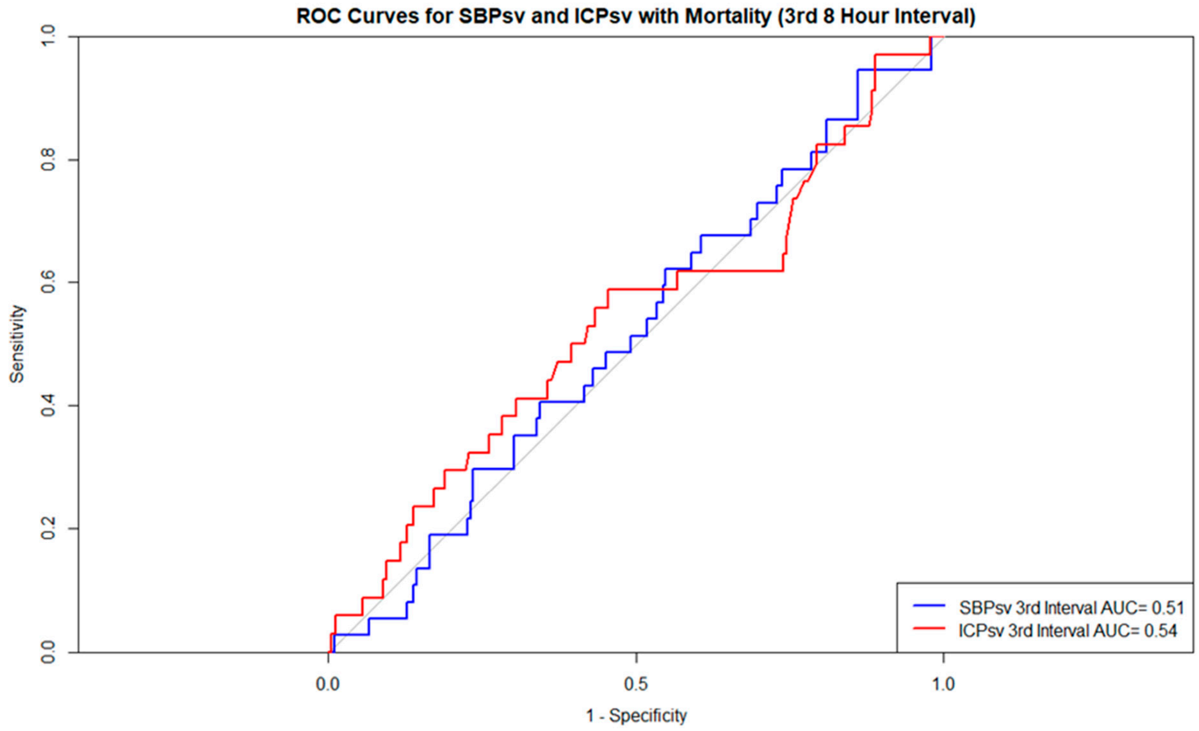

AUC = area under the curve; ICPsv = successive variation of intracranial pressure; ROC = receiver operating characteristic; SBPsv = successive variation of systolic blood pressure.

Supplementary Figure S2. ROC Curves for Discharge Home 2nd and 3rd 8-hour interval

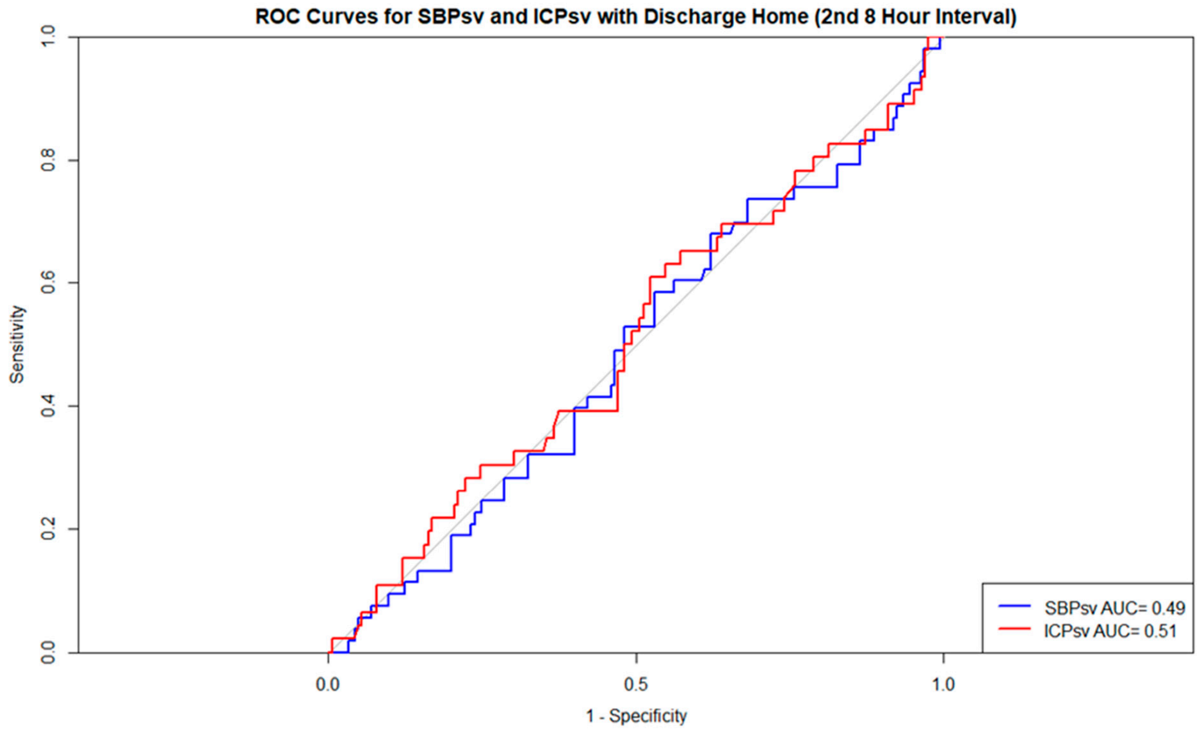

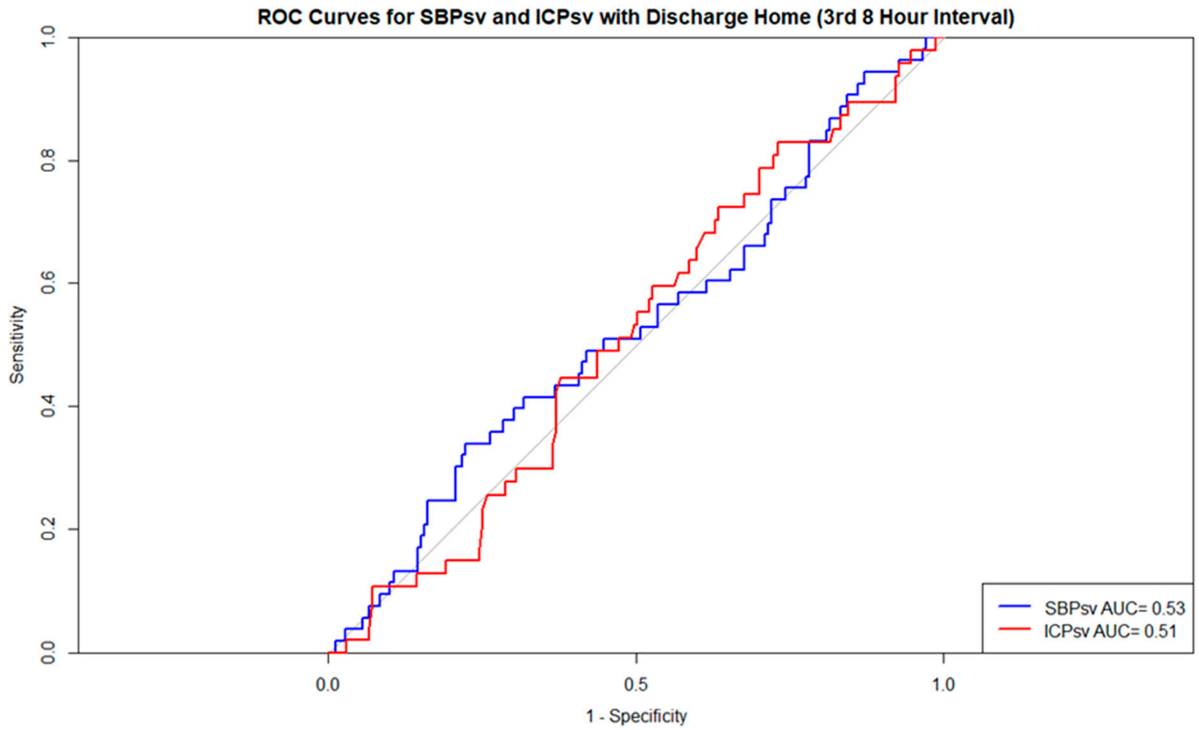

AUC = area under the curve; ICPsv = successive variation of intracranial pressure; ROC = receiver operating characteristic; SBPsv = successive variation of systolic blood pressure.
